# Effects of dietary supplementation of mannan-oligosaccharide on virus shedding in avian influenza (H_9_N_2_) challenged broilers

**Published:** 2016

**Authors:** T. Akhtar, G. Ara, N. Ali, F. ud Din Mufti, M. Imran Khan

**Affiliations:** 1 MPhil/Ms in Physiology, Department of Physiology, University of Veterinary and Animal Sciences, Lahore-5400, Punjab, Pakistan;; 2 MPhil/Ms in Genetic Engineering and Biotechnology, Institute of Biotechnology and Genetic Engineering, The University of Agriculture, Peshawar-25120, KPK, Pakistan;; 3 MPhil/Ms in Livestock Management, Department of Livestock Management, Faculty of Animal Husbandry and Veterinary Sciences, The University of Agriculture, Peshawar-25120, KPK, Pakistan;; 4 MPhil/Ms in Biotechnology, Department of Biotechnology, Quaid-i-Azam University, Islamabad, Pakistan;; 5 Department of Animal Health, Faculty of Animal Husbandry and Veterinary Sciences, The University of Agriculture, Peshawar-25120, KPK, Pakistan

**Keywords:** Avian influenza, Broiler, MOS, SAF-Mannan

## Abstract

Avian influenza (AI) is a highly contagious disease causing significant economic losses worldwide. The aim of this study is to evaluate the effect of mannan-oligosaccharide (MOS) on tracheal and cloacal virus shedding in AI challenged broilers and contamination of environment with H_9_N_2_. A total of 300 1-day-old-broiler chicks were randomly divided into 3 groups (A, B and C) and supplemented 0.2, 0.5 and 0.0% MOS, respectively in NRC recommended diet for 36 days. On day 21 the groups were further split into two sub groups A+ve, A-ve, B+ve, B-ve, C+ve and C-ve with 5 replicates each. The positive groups were shifted to remote sheds and were challenged intranasally with 0.1 ml of reference virus (AIV; Pk-UDL/01/08 H_9_N_2_) with EID_50_ = 10^-6.66^. Treatment reduces (P<0.05) cloacal virus shedding from day 24 to 26 and 28 to 32. Tracheal virus shedding was lower (P<0.05) on days 25-26 and 28-30 in treatment groups. Day 27 showed highest (P>0.05) virus shedding in all groups. However the reduction of viral shedding is faster in treatment groups and showed no virus shedding on day 32. Maternal antibody titer against AI showed a declining pattern but MOS influenced (P<0.05) the titer in treated groups. Hence the use of MOS may constitute a novel and effective plausible alternative that reduces the spread of disease by decreasing virus shedding and contamination of environment from AIV (H_9_N_2_) infection in poultry.

## Introduction

Avian influenza (AI) virus is a member of family Orthomyxoviridae. The infection with a highly virulent virus is characterized by sudden onset of high mortality together with respiratory signs, sinusitis, excessive lacrimation, rales, cessation of egg laying, cyanosis especially on comb and wattles, edema of head and face, diarrhea and weight loss with fatal outcome (Noble, 1982 ▶) and enormous economic losses (Bhatti, 1995 ▶). Extracellular host proteases (trypsin-like enzymes) are required for cleavage of viruses and hence its replication is restricted to intestine (Poorbaghi et al., 2013 ▶) and respiratory tracts (Kawase et al., 2010 ▶) where such enzymes are found. The ability of virus to spread is related to the amount of virus released by the respiratory or intestinal route (Youn et al., 2012 ▶).

Some strains of live yeast are effective in reducing intestinal pathogen counts (Saf-Agric Inc., 2007 ▶). Mannan-oligosaccharide (MOS) obtained from mannans present on the cell wall of a yeast *Saccharomyces cerevisiae* (Spring et al., 2000 ▶) is a well-recognized prebiotic that acts as a high affinity ligand and offers a competitive binding site for the microorganisms. Different studies showed that pathogenic microbes having mannose-specific fimbriae can bind via type-1-fimbriae to mannose which reduces the risk of pathogen microbes such as *Salmonella*, *Escherichia* *coli* and *Clostridia* in the intestinal tract (Oyofo et al., 1984 ▶). Secondly, the fore-gut does not possess the enzymes required for the breakdown of prebiotics, resultantly they reach the hind gut intact (Strickling et al., 2000 ▶) where they are utilized by the beneficial microbiota (Qaisrani et al., 2015 ▶) to produce volatile fatty acids (Yang et al., 2007 ▶), ultimately the microenvironment of brush border becomes acidic and minimizes the chances of pathogenic growth and colonization for better reproduction (Havenaar and Huis In’t Veld, 1992 ▶).

It is assumed that prebiotics have effects on the immune system, as it stabilizes the intestine by enhancing the gut microflora (Glenn and Roberfroid, 1995 ▶; Ribeiro et al., 2007 ▶) which can modify host innate and acquired immune responses (Schley and Field, 2002 ▶; Oliveira et al., 2009 ▶) by which they can minimize the deleterious effects of respiratory and gut infections and hence reduce the contamination of environment and spread of disease. The current trial was therefore designed to assess the effect of MOS on virus shedding and so environment contamination from tracheal and intestinal route in AI challenged broilers.

## Materials and Methods

**Experimental design**

A total of 310 commercial 1-day-old Arbor Acre broilers were procured from a reputable firm (Ani Chicks (Pvt.) Limited) and transferred to experimental shed of University of Veterinary and Animal Sciences, Lahore. Chicks were individually marked using cotton tape wrapped around the wing and was replaced thrice with increase in growth. Ten chicks were slaughtered on day 1 to collect blood serum for presence of maternal antibodies against *Mycoplasma gallisepticum*, Newcastle disease virus and AI virus subtype H_9_. The remaining chicks were randomly divided (completely randomized design) into three equal groups (A, B and C) with ten replicates (n=20). The treated groups of A and B were supplemented with 0.2% and 0.5% MOS, respectively from commercially available product SAF-Mannan (S. I. LeSaffre, Cedex, France) in NRC (2001) ▶ recommended diet (Table 1) with free access to water up to thirty-six days of age. The chicks were weighed and recorded at day one and were raised as per standard management conditions. Chicks were vaccinated against Newcastle 1,200 mg; Se, 8 mg; Co, 20 mg; I, 40 mg; vitamin A, 200,000 IU; vitamin D3, 80,000 IU; vitamin E, 1,072 IU; vitamin K3, 34 mg; ascorbic acid, 1,300 mg; thiamine, 35 mg; riboflavin, 135 mg; niacin, 1,340 mg; vitamin B6, 100 mg; folic acid, 34 mg; vitamin B12, 670 μg; and biotin, 3,350 μg disease at the age of 2 and 11 days and Infectious Bursal Disease on day 6 and 16 through oral route and were reared on wood shaving litter of 2 inch thickness. Temperature and relative humidity on day one was maintained at 95°F and 65 ± 5%, respectively. Temperature was decreased 5°F per week until it reached at 70-75°F and relative humidity was maintained. Chicks were provided 40-60 lux intensity of light for 23 h and 1 h darkness throughout the experiment period. Groups A, B and C were further divided into 2 subgroups, A+ve, A-ve, B+ve, B-ve, C+ve and C-ve, respectively on day 21 with 5 replicates each (n=10). The blood samples were collected on day 1, 7, 14 and 21 for monitoring the serum AI antibody titer against AI virus subtype H_9_ using Haemagglutination inhibition assay as described by Alexander and Chettle (1977) ▶. The university animal care and ethical committee approved all the procedures in this study.

**Table 1:** Ingredient (%) and nutritive value of a basal diet for broilers

Ingredients^*^	Starter (%)	Finisher (%)
Corn	59.81	54.5
Soybean meal 48%	32.0	36.1
Soybean oil	4.33	5.6
Monocalcium phosphate	1.45	1.3
Limestone	1.12	1.3
Premix^1^	1.00	1.0
Salt	0.07	0.07
Magnesium oxide	0.05	0.05
DL-Methionine	0.13	0.03
L-Lysine HCl	0.04	0.05
Total	100.0	100.0
**Nutrient value**		
DM (%)	87	88
Calculated ME (Kcal/kg)	2750	2850
CP (%)	19.6	18.5
Crude fat (%)	2.16	2.35
Crude fiber (%)	1.26	1.80
Total ash (%)	5.77	5.40

* Standard constituents of commercial feed (NRC, 2001).

1 Vitamin-mineral premix (each kg contained): Ca, 195 g; K, 70 g; Na, 18 g; Mg, 6 g; Zn, 1,200 mg; Fe, 2,000 mg; Cu, 400 mg; Mn,

**Avian influenza H**_9_ **virus challenge**

The reference stock AI virus (Pk-UDL/01/08 H_9_N_2_) for challenge was obtained from University Diagnostic Lab. Before challenge the groups A+ve, B+ve and C+ve were shifted to remote shed and challenged intranasally with 0.1 ml of reference virus (AIV; Pk-UDL/01/08 H_9_N_2_) with mean egg infective dose (EID_50_) = 10^-6.66^.

**Calculation of mean EID**_50_

*Propagation of virus in embryonated hen eggs*

The reference virus was propagated in 9-11 day-old embryonated eggs (Rickard et al., 1944 ▶). The eggs were candled after 24 and 48 h for any mortality. The eggs were transferred to refrigerator for an overnight chilling to clot blood so as to facilitate the harvesting of the allanto-amniotic fluid (AAF).

*Harvesting AAF*

AAF was cultured on blood agar medium to confirm its sterility and agar plates were incubated overnight at 37°C. The aliquots of sterile AAF were made in microfuge tubes and stored at -80°C till further use.

*Calculation of EID*_50_ *of AAF*

EID_50_ was calculated by the procedure as described by Reed and Muench (1938) ▶.

**Virus shedding**

*Tracheal and cloacal swabs*

The tracheal and cloacal swabs of live chicks were collected using universal viral transport swabs (BD, USA) from days 22 to 28 and then at alternate days i.e., day 30, 32, 34 and 36 to detect levels of virus shedding. The swabs were put back into a vial containing 3 ml of 15% brain heart infusion medium containing antibiotics (1000 µL/ml Gentamycin + 10,000 IU/ml Penicillin G + 20 µL/ml Amphotericin B).

*Determining EID*_50_ *of the cloacal and tracheal swabs*

One aliquot each of cloacal and tracheal swabs was used to calculate EID_50_ of virus suspension (Reed and Muench, 1938 ▶).

**Statistical analysis**

Normal distribution of data was analyzed by Kolmogorov-Smirnov test. Treatment effect was analyzed using one-way analysis of variance by using commercially available statistical package SPSS (version 18.0, for Windows; SPSS, Chicago, IL). Repeated measure analysis of variance was applied to analyze virus shedding across time in the same group (Steel and Dickey, 1997 ▶). Duncan’s multiple range test was subjected to determine group differences (Duncan, 1955 ▶). Probability values <0.05 were accepted as significant.

## Results

**Fig. 1:**
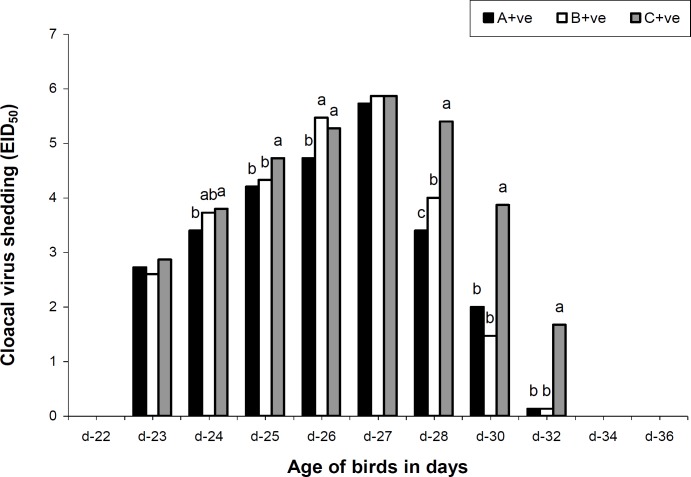
Mean cloacal virus shedding of control and MOS supplemented groups of broilers challenged with avian influenza virus (H_9_N_2_). Data coded as EID_50_ 10^0^=0-1; EID_50_ 10^-1.60^=2; EID_50_ 10^-2.60^=3; EID_50_ 10^-3.20^=4; EID_50_ 10^-3.60^=5; EID_50_ 10^-4.23^=6. ^a-c^ Mean with different superscripts are significantly different from each other (P<0.05). Whereas A+ve (0.2% MOS + AI challenge); B+ve (0.5% MOS + AI challenge) and C+ve (0.0% MOS + AI challenge

**Fig. 2:**
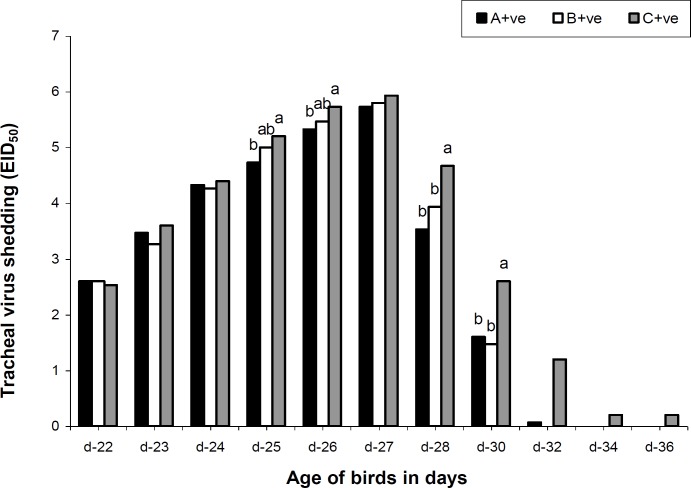
Mean tracheal virus shedding of control and MOS supplemented groups of broilers challenged with avian influenza virus (H_9_N_2_). Data coded as EID_50_ 10^0^=0-1; EID_50_ 10^-1.60^=2; EID_50_ 10^-2.60^=3; EID_50_ 10^-3.20^=4; EID_50_ 10^-3.60^=5; EID_50_ 10^-4.23^=6. ^a-c^ Mean with different superscripts are significantly different from each other (P<0.05). Whereas A+ve (0.2% MOS + AI challenge); B+ve (0.5% MOS + AI challenge) and C+ve (0.0% MOS + AI challenge

**Fig. 3:**
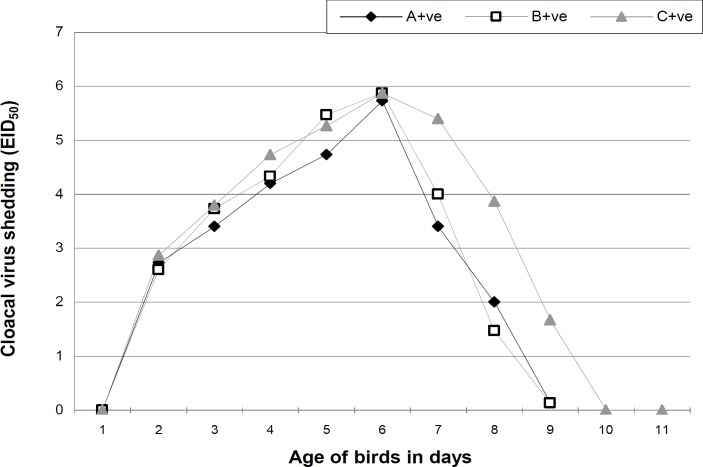
Mean cloacal virus shedding across time of control and MOS supplemented groups of broilers challenged with avian influenza virus (H_9_N_2_). A+ve (0.2% MOS + AI challenge); B+ve (0.5% MOS + AI challenge) and C+ve (0.0% MOS + AI challenge

**Fig. 4:**
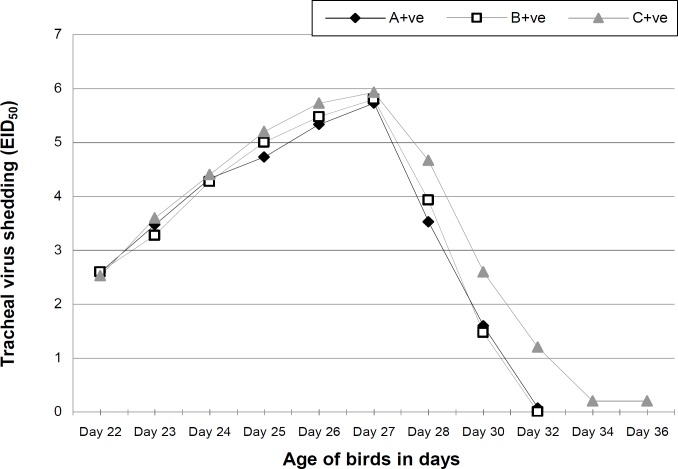
Mean tracheal virus shedding across time of control and MOS supplemented groups of broilers challenged with avian influenza virus (H_9_N_2_). A+ve (0.2% MOS + AI challenge); B+ve (0.5% MOS + AI challenge) and C+ve (0.0% MOS + AI challenge

Maternal AI antibody titer was highest on day 1 and showed a declining pattern in all groups. However, the titer was higher in treatment groups on day 7, 14 and 21 but no longer protective (Table 2). The antibody titer increased to as high as 101.4, 227.5 and 801.4 GMT in the 4th week of age among groups A, B and C, respectively. The cloacal virus shedding was the same in all groups on day 23 and 27. However, from day 24 to 26 and 28 to 32, it was noted that the virus shedding from cloacal route was significantly lower in treatment groups. No virus shedding from cloacal route was observed on day 32 (Fig. 1). On day 22, 23, 24 and 27 the tracheal viral shedding was the same (P>0.05) in all groups, however from day 25 to 26 and 28 to 30 the tracheal virus shedding was significantly higher in control group (Fig. 2). Cloacal virus shedding increases from day 23 to 27 and then declines sharply (Fig. 3). The same trend was observed in tracheal virus shedding (Fig. 4). Furthermore, day 27 showed peak (P<0.05) virus shedding in all groups. However, the reduction was fast (P<0.05) in treatment groups (Figs. 3 and 4).

**Table 2:** Mean log_2_ AI antibody titers with standard errors of broiler chicken

Day	A+ve	B+ve	C+ve
1	3.3 ± 0.17	2.8 ± 0.38	3.0 ± 0.37
7	1.9 ± 0.43^a^	1.7 ± 0.17^a^	0.7 ± 0.26^b^
14	1.4 ± 0.30^a^	1.2 ± 0.23^a^	0.2 ± 0.13^b^
21	0.6 ± 0.12^a^	0.8 ± 0.13^a^	0.0 ± 0.00^b^

Mean±SEM (n=10) within a row lacking a common superscript differ significantly from one another (P<0.05)

## Discussion

Several earlier experiments revealed that MOS influence antibody titer (Cotter et al., 2000 ▶; Raju and Devegowda, 2002 ▶; Janardhana et al., 2009 ▶; Silva et al., 2009 ▶). This indicates that the supplemented prebiotic has a positive effect on immune response of birds (Shashidhara and Devegowda, 2003 ▶; Lourenço et al., 2016 ▶) and decreases severity of AI (Tohid et al., 2010 ▶; Shahir et al., 2014 ▶) and faecal shedding (Youn *et al*., 2012). The results of our findings are in accordance with the study of Poorbaghi et al. (2013 ▶) who investigated inulin-based probiotic decreased faecal shedding of H_9_N_2_ AIV in non-vaccinated groups. Similarly, Fang et al., (2009) ▶ reported a dose dependent relationship of faecal rotavirus shedding and lactobacillus. Our study is also in agreement with the study of Youn et al. (2012) ▶, who demonstrated that less number of indirect contact chicken shed AI virus from gastro-intestinal tract upon intranasal administration of selected lactobacilli, CJL (Lactobacillus fermentum CJL-112). Furthermore, virus shedding from respiratory tract was also reduced in challenged and direct contact chickens. It is suggested that due to presence of exogenous CJL which is supposed to inhibit replication of AIV H_9_N_2_ in respiratory tract and hence provide protection against horizontal spread of AIV H_9_N_2_ and environmental contamination. Most importantly, probiotics have been shown to regulate the expression of genes related to innate immune-mediated cytokine responses in the intestinal (Ganguli et al., 2013 ▶) and also the respiratory mucosa, creating an anti-inflammatory milieu and thus modulating beneficially respiratory mucosal antibacterial and antiviral immunity. Our findings are also in agreement with the study of Qiao et al., (2001) ▶ who reported the clinical diarrhea and viral shedding were significantly delayed in probiotic and bifidobacteria supplemented mice challenged with Rhesus Rotavirus.

 In conclusion, we demonstrated that the dietary supplementation of MOS markedly diminished rep-lication and shedding of H_9_N_2_ and morbidity in broilers horizontally infected with H_9_N_2_ AIV. Hence the use of MOS may constitute a novel and effective plausible alternative that reduces environmental contamination and spread of H_9_N_2_ from infected birds.

## Conflict of interest

 No conflict of interest declared.
